# Reward-Related Attentional Bias and Adolescent Substance Use: A Prognostic Relationship?

**DOI:** 10.1371/journal.pone.0121058

**Published:** 2015-03-27

**Authors:** Madelon E. van Hemel-Ruiter, Peter J. de Jong, Brian D. Ostafin, Albertine J. Oldehinkel

**Affiliations:** 1 Department of Clinical Psychology and Experimental Psychopathology, University of Groningen, Groningen, the Netherlands; 2 Interdisciplinary Center Psychopathology and Emotion regulation, University of Groningen, University Medical Center Groningen, Groningen, the Netherlands; Washington State University College of Veterinary Medicine, UNITED STATES

## Abstract

Current cognitive-motivational addiction theories propose that prioritizing appetitive, reward-related information (attentional bias) plays a vital role in substance abuse behavior. Previous cross-sectional research has shown that adolescent substance use is related to reward-related attentional biases. The present study was designed to extend these findings by testing whether these reward biases have predictive value for adolescent substance use at three-year follow-up. Participants (N = 657, mean age = 16.2 yrs at baseline) were a sub-sample of Tracking Adolescents’ Individual Lives Survey (TRAILS), a large longitudinal community cohort study. We used a spatial orienting task as a behavioral index of appetitive-related attentional processes at baseline and a substance use questionnaire at both baseline and three years follow-up. Bivariate correlational analyses showed that enhanced attentional engagement with cues that predicted potential reward and nonpunishment was positively associated with substance use (alcohol, tobacco, and cannabis) three years later. However, reward bias was not predictive of changes in substance use. A post-hoc analysis in a selection of adolescents who started using illicit drugs (other than cannabis) in the follow-up period demonstrated that stronger baseline attentional engagement toward cues of nonpunishment was related to a higher level of illicit drug use three years later. The finding that reward bias was not predictive for the increase in substance use in adolescents who already started using substances at baseline, but did show prognostic value in adolescents who initiated drug use in between baseline and follow-up suggests that appetitive bias might be especially important in the initiation stages of adolescent substance use.

## Introduction

Substance abuse and dependence are major problems at both the individual and the societal level. Substance use often starts in adolescence [1], and it has been found that a younger age at the onset of use is a risk factor for later dependence and abuse [2–5], with the greatest risk for youth beginning to use in the teenage years [2]. Germane to this, there is growing evidence that appetitive, reward-related attentional bias plays a role in substance (mis)use. Through conditioning, substance-related stimuli can become cues of reward (or relief), which then attract attention. Once the cues have been noticed, they may elicit craving and behavioral dispositions to approach and consume the drug [6–8]. There is evidence that attentional bias toward specific drug cues is related to drug behavior [6,9,10]. Attentional bias toward general cues of reward should similarly increase the likelihood of drug cues attracting attention (given their status as cues of reward). That is, people who respond strongly to general reward cues might be more willing to try and use more substances, than those who are less attentive to cues of reward. Support for this idea is found in previous studies that have demonstrated that attentional biases for general reward cues are positively related to alcohol use in students [11], and to substance use in (young) adolescents [12].

These previous studies on the relationship between substance use and attentional bias toward reward have used the Spatial Orienting Task [13] as a measure of attentional processes toward cues of general reward [11,12]. This task was developed to explore to what extent people direct and hold their attention to places where a potential reward or prevention of punishment (i.e., nonpunishment) are expected. In terms of Gray’s Reinforcement Sensitivity Theory, the Behavioral Approach System (BAS) is proposed to be responsible for organizing behavior in response to appetitive stimuli, including reward and nonpunishment [14,15]. Attentional biases as indexed by the spatial orienting task (SOT) have been linked to reward and punishment related processes, suggesting that this task is useful for assessing prioritized processing of both positive and negative incentives [11,16,17]. Although previous research has found a consistent link between adolescent substance use and high self-reported reward sensitivity [18–21], relatively little research has examined whether behavioral measures of reward and punishment sensitivity are related to substance use.

Using a SOT as an index of reward-related attentional bias, it was previously found that adolescents who attended more quickly to places where a reward or nonpunishment was expected reported a higher level of alcohol, tobacco, and cannabis use [12]. Although these earlier findings are consistent with the idea that heightened reward-related attentional bias plays a role in substance misuse, the cross-sectional design of that previous study prevents the ability to make directional inferences regarding the relationship between reward-related attentional biases and substance use. Therefore, the present study used a longitudinal approach to examine whether a general reward-related attentional bias would show a prospective relationship with adolescent substance use at a three-year follow-up, and whether reward bias would also predict the increase in substance use within this follow-up period. First, we tested the hypothesis that an attentional bias toward cues of reward and nonpunishment would be associated with high levels of prospective substance use. In line with the previous cross-sectional findings, we expected this bias to emerge as an enhanced engagement toward both rewarding cues and cues of nonpunishment. To enable comparison with our previous cross-sectional findings, we again focused on the prediction of alcohol, tobacco, and cannabis use. Second, we tested if reward-related attentional bias has predictive value for prospective substance use over and above initial substance use during the baseline assessment. If reward-related attentional bias would indeed precede an increase in substance use, this would point to reward-related attentional bias as a promising focus of preventive interventions.

## Materials and Methods

### Participants and Recruitment

For the current study we used the same sample as van Hemel-Ruiter et al. [12]. Participants were a sub-sample of Tracking Adolescents’ Individual Lives Survey (TRAILS), a large prospective population study of Dutch adolescents with bi or triennial measurements from age 11 to at least age 25. This cohort of 2230 adolescents (Baseline: mean age = 11.09 years, SD = 0.56, 50.8% female, response rate 76%) was recruited via primary schools in five northern municipalities (including urban and rural areas) and constituted of 64% of all children born between October 1989 and September 1990 (first three municipalities) or October 1990 and September 1991 (last two municipalities) in these areas (for more details [22,23]). The present study reports data from the third (T3; from 2005 to 2007) and fourth (T4; from 2008 to 2010) assessment wave, with the fourth wave being three years following the third. In T3 a total of 1816 (81% of initial sample, mean age T3 = 16.3, range = 14.7–18.7), and in T4, a total of 1881 (84% of initial sample, mean age T4 = 19.1, range = 18.0–20.9) adolescents participated [24]. For reasons of feasibility and costs, a focus cohort of 744 adolescents was invited to perform a series of laboratory tasks on top of the usual assessments, of which 715 (96% of initial sample) agreed to participate. Adolescents with a high risk of mental health problems had a greater chance of being selected for the experimental session. High risk was defined based on temperament (high frustration and fearfulness, low effortful control), lifetime parental psychopathology (depression, anxiety, addiction, antisocial behavior, psychoses), and living in a single-parent family. In total, 66% of the focus cohort had at least one of these risk factors. The remaining 34% were randomly selected from the low-risk TRAILS participants. Hence, the focus cohort still represented the whole range of problems seen in a normal population of adolescents, which made it possible to represent the distribution in the total TRAILS sample by means of sampling weights (for more detailed information on the selection procedure and response rates within each stratum, see [12]). Around 92% of this focus cohort (*n* = 654) had completed both the Spatial Orienting Task at T3 and the Substance Use Questionnaire (SUQ) at T3 and T4. As a result of the exclusion of 61 participants, who carried different weights, the use of this weighting procedure resulted in a deviant final weighted sample size of 657. Due to a small percentage of missing values (<0.5%) we imputed the data-set by conducting mean substitution. Descriptive statistics of the final imputed sample in T3 and T4 (weighted estimates) are presented in Table 1.

**Table 1:** Sample Characteristics (N = 657^a^).

	Baseline (T3)	3-year follow-up (T4)
Variable		
Female Gender	52.3%	52.3%
Age (mean [SD])	16.1 [0.59]	19.0 [0.54]
Servings alcohol/week previous month^b^ (median [range])	4.00 [0–69.5]	6.30 [0–73.5]
Cigarettes/week previous month (median [range])	0.00 [0.0–210]	0.00 [0–224]
Frequency of cannabis use over previous month (median [range])	0.00 [0.0–40.0]	0.00 [0–40.0]
Lifetime user of illicit drugs (other than cannabis)	5.5%	13.6%
Lifetime abstainer of alcohol, tobacco and drugs	6.1%	3.1%

^a^ The sample size reported reflects the weighted sample size.

^b^ One serving of alcohol contains approximately 11 ml of pure alcohol.

The experimental protocol and consent procedure were approved by the Central (Dutch) Committee on Research Involving Human Subjects (CCMO). All participating adolescents and their parents gave written informed consent.

### Procedure

#### Laboratory Behavioral Assessment

As an index of attentional bias for appetitive stimuli we used the Spatial Orienting Task [16]. The SOT was the first computer task of a larger set of experimental tests, included in the third assessment wave. The test assistants received extensive training in order to optimize standardization of the experimental session. Participants were tested on weekdays, in a sound-attenuating room with blinded windows at selected locations in the participants’ town of residence.

#### Spatial Orienting Task

The task was presented on a Philips Brilliance 190 P monitor controlled by an Intel Pentium 4 CPU computer using E-prime software version 1.1 (Psychology Software Tools Inc, Pittsburgh, Pennsylvania). Participants were seated 50 cm away from the screen and responses were collected on the computer’s keyboard.

#### Task description

In collaboration with Derryberry and Reed, we programmed a SOT that was virtually identical to their original task [16]. The task consisted of four positive and four negative blocks of trials (games), which alternated in sets of two, starting with two positive games. On positive blocks, participants gained 10 points for fast responses, and did not gain points for slow responses (definitions of *fast* and *slow* are given below). On negative blocks, participants lost 10 points for slow responses, and did not lose points for fast responses. Regardless of the block, ten points were lost for inaccurate responses. To enhance motivation, participants were informed that those with the highest scores in the positive games would win a price, while extremely low scores in the negative games could result in having to do the task again, until performance would be good enough. Therefore, the feedback on scores was used to enhance motivation to play the game as fast and accurate as possible.

#### Stimuli

Throughout each game, two vertical black bars were displayed against a white background, which marked the location of the cues and targets. Participants were instructed to fixate on the score which was presented in black at the screen’s center. The score was updated after each response (see below) and remained on the screen throughout the trial. Each trial began with turning the fixation score off for 200 ms and then back on for 250 ms. Next, a cue arrow replaced one of the two vertical black bars. After a delay of 250 (*short delay*) or 500 ms (*long delay*), a target appeared. The target was a small vertical gray rectangle centered within the cue arrow (*cued target)* or within the vertical black bar on the opposite side of the fixation score (*uncued target*). Participants were told that a blue up-arrow (*easy cue)* signaled that a target appearing in that location (cued) would be ‘easy’ (i.e., own mean RT + 0.55 SD to react) and result in a sufficiently fast response about 75% of the time, whereas a target in the uncued bar’s position would be ‘hard’ (i.e., own mean RT − 0.55 SD to react), that is, resulting in a too slow response about 75% of the time. A red down-arrow (*hard cue*) indicated that a cued target would be ‘hard’ (the response would be too slow 75% of the time) and an uncued target ‘easy’ (the response would be sufficiently fast 75% of the time). Additionally, they were informed that the cue would also indicate the probable location of the target, with 2/3 of the targets appearing in the cued location, and that occasionally no target would appear (*catch trials*). Participants were instructed to press the ‘b’ key as soon as they detected the target. Pressing the key before the target appeared or when no target appeared resulted in a loss of 10 points. Each block consisted of 32 cued, 16 uncued, and 8 catch trials, randomized across subjects (i.e., for every subject trials were presented in an independent order). Five hundred ms after the response (or 1 s following the delay interval on catch trials), the cue arrow and target were removed by reinstating the two black bars, and a feedback signal was presented below the central score. Feedback consisted of the same arrows as used for the cues. A blue up-arrow indicated a fast response or (accurate) non-response on catch trials. A red down-arrow signaled a slow response or (inappropriate) response on catch trials. After a delay of 250 ms, the score was updated (if changed). After a randomly selected ITI of 500 or 1000 ms, the next trial began by removing the feedback signal and blanking the score for 200 ms (see [12] for more detailed descriptions of this task).

#### Feedback computation

At the end of each game, the participant’s median RT and standard deviation were computed to calculate cut-offs for fast and slow responses on the next game of the same type (positive or negative). Consistent with the previous work of Derryberry and Reed, for easy targets, the response was labeled as fast if the RT was less than the median plus 0.55 times the standard deviation. For hard targets, a response was treated as fast if the RT was less than the median minus 0.55 times the standard deviation. If RTs equaled or exceeded these cut-offs, they were treated as slow. Because RTs tend to be about 25 ms slower after short delays, 12 ms were added to the cut-off for short-delay trials and subtracted for long-delay targets (see [12] for more detailed task description, also see [16]. Because the response-window was adapted on-line on the basis of the participant’s individual performance, there were no participants with extremely low scores.

#### Self-Reported Substance Use

Measures of alcohol, tobacco, cannabis, and other drug use were part of a larger self-report survey. At the third assessment wave, participants filled in these questionnaires at school, which was supervised by test assistants (see [25]). At the fourth assessment wave a web-based survey method was used (see [24]). Substance use was calculated on quantity and frequency items of alcohol use (seven items, e.g., At how many days did you drink alcohol last week, How many times did you drink alcohol in your lifetime?), tobacco use (three items, e.g., Did you ever smoke, even if it was just one cigarette or a few drafts?), and cannabis use (three items, e.g., How many times did you use weed or hash in the last four weeks?) (See S1 Table for an overview of all substance use questions). Drug use other than cannabis was left out of the substance use variable, to enable comparison with our previous cross-sectional findings [12]. Because of their different scaling, standardized scores were used to calculate measures for alcohol, and cannabis use. Finally, as an index of general substance use, we used the means of the alcohol, tobacco, and cannabis measures to calculate a substance use measure (see the S1 Table for the Cronbach’s alphas).

### Data Reduction and Analysis

The SOT reaction time data were analyzed following Derryberry and Reed [16]. Trials with reaction times below 125 ms (probable anticipations) and above 1000 ms (probable distractions) were removed. Mean reaction times for correct responses are reported in Table 2.

**Table 2:** Mean score reaction times (M in ms) and standard deviations (sd) of SOT scores (N = 657ª).

Type of game	Short Delay				Long Delay			
	Cued		Uncued		Cued		Uncued	
	Easy	Hard	Easy	Hard	Easy	Hard	Easy	Hard
	M(sd)	M(sd)	M(sd)	M(sd)	M(sd)	M(sd)	M(sd)	M(sd)
Positive	335(41)	366(47)	467(89)	470(89)	341(576)	378(67)	384(77)	377(73)
Negative	329(45)	357(52)	455(88)	458(92)	331(58)	365(67)	381(81)	373(77)

Note: SOT = Spatial Orienting Task.

^a^ The sample size reported reflects the weighted sample size.

Further, we computed the engagement and disengagement scores. That is, from positive game RT scores engagement towards reward was calculated by subtracting RT cued blue trials from RT cued red trials in the positive games, whereas difficulty to disengage from reward was calculated by subtracting RT uncued red trials from uncued blue trials. From negative game RT scores engagement towards non-loss was calculated by subtracting RT cued blue trials from RT cued red trials in the positive games, whereas difficulty to disengage from non-loss was calculated by subtracting RT uncued red trials from uncued blue trials. Hence, attentional bias for reward was represented in the positive games as both (1) a faster engagement toward cues of expected gain (blue arrow acting as correct cue for target; *cued blue trials*) than toward cues of expected non-gain (red arrows acting as correct cue for target; *cued red trials*) and (2) a slower disengagement from expected gain (blue arrow acting as incorrect cue for target; *uncued blue trials*) than from expected non-gain (red arrows acting as incorrect cue for target; *uncued red trials*). Analogously, attentional bias for nonpunishment was represented in the negative games, by both (1) a faster engagement toward cues of expected non-loss (blue arrows acting as correct cue for target; *cued blue* trials) than toward cues of expected loss (red arrows acting as correct cue for target; *cued red trials*) and (2) slower disengagement from expected non-loss (blue arrow acting as incorrect cue for target; *uncued blue trials*) than from expected loss (red arrows acting as incorrect cue for target; *uncued red trials*). All scores were separately calculated for short-delay and long-delay trials (i.e., when there was less or more opportunity for voluntary control processes to regulate attention).

To investigate the relationship between attentional biases and prospective substance use we first performed a bivariate correlational analysis, which included prospective substance use, gender, age, all eight engagement and disengagement scores, and baseline substance use. Next, to test the unique predictive contribution of all engagement and disengagement scores, and baseline substance use in the prediction of substance use three years later we performed a stepwise hierarchical regression. Step 1 included age, gender, and baseline substance use and step 2 included the eight engagement and disengagement scores.

## Results

### Reliability

Split-half correlations with Spearman-Brown corrections demonstrated substantial internal consistency for SOT mean RT’s (*r*s = 0.54–0.79) whilst attentional bias scores showed only low to moderate internal consistency (*r*s = 0.00 to 0.34).

### Correlation analysis

Our first hypothesis was that prospective substance use would be predicted by baseline engagement toward both rewarding cues and cues of nonpunishment. Table 3 shows that engagement toward rewarding cues (but only for long-delay trials) and engagement toward cues of nonpunishment (but only for short-delay trials) correlated weakly to prospective substance use. Additionally, prospective substance use was weakly correlated with gender, and very strongly with baseline substance use (*r* = 0.72, *p* < 0.01).

**Table 3:** Bivariate correlations of attentional bias scores and substance use (N = 657ª).

		1	2	3	4	5	6	7	8	9	10	11	12
1	Prospective substance use	-											
2	Gender ^b^	0.12**	-										
3	Age at follow-up	0.07	0.02	-									
4	Attentional engagement toward reward (short-delay)	0.04	0.03	−0.07	-								
5	Difficulty disengaging from reward (short-delay)	−0.07	−0.07*	−0.01	−0.04	-							
6	Attentional engagement toward nonpunishment (short-delay)	0.11**	0.03	−0.09*	0.29**	−0.05	-						
7	Difficulty disengaging from nonpunishment (short-delay)	0.00	0.05	0.04	0.01	0.03	−0.07	-					
8	Attentional engagement toward reward (long-delay)	0.11**	0.00	-0.02	0.24**	0.01	0.13**	−0.06	-				
9	Difficulty disengaging from reward (long-delay)	−0.01	0.00	0.00	−0.02	0.04	−0.09*	−0.03	−0.01	-			
10	Attentional engagement toward nonpunishment (long-delay)	0.06	0.03	−0.05	0.25**	0.04	0.20**	0.04	0.20**	0.01	-		
11	Difficulty disengaging from nonpunishment (long-delay)	−0.03	−0.01	−0.04	−0.01	0.04	−0.08*	0.00	−0.08*	0.04	0.00	-	
12	Baseline substance use	0.72**	0.02	0.10**	0.10*	−0.04	0.13**	0.02	0.14**	−0.02	0.07	-0.02	-

* p < 0.05.

** p < 0.01.

^a^ The sample size reported reflects the weighted sample size.

^b^ 0 = Female, 1 = Male.

### Regression analysis

The hierarchical regression analysis showed gender, and baseline substance use predicted unique variance of adolescent substance use three years later, but the engagement and disengagement scores showed no predictive validity on top of these variables. Overall, the full model explained 52% (*R²* adjusted = 0.52, *F*(11, 645) = 64.48, *p* <0.001) of all variance (Table 4). The model showed that male adolescents showed a larger increase in substance use than female adolescents, yet reward related attentional biases showed no predictive value for future substance use over three years follow-up.

**Table 4:** Hierarchical regression model for variables explaining prospective substance use (N = 657ª).

Variable		Beta	T	R² Change
Step 1				
	(Constant)		−0.10	
	Gender ^b^	0.11	3.86**	
	Age	0.00	−0.00	
	Baseline Substance Use	0.71	26.13**	0.52
Step 2				
	(Constant)		−0.04	
	Gender ^b^	0.10	3.80**	
	Age	0.00	−0.06	
	Baseline Substance Use	0.71	25.50**	
	Engagement toward reward (short-delay)	−0.05	−1.64	
	Engagement toward nonpunishment (short-delay)	0.02	0.53	
	Engagement toward reward (long-delay)	0.02	0.54	
	Engagement toward nonpunishment (long-delay)	0.02	0.68	
	Disengagement from reward (short-delay)	−0.03	−1.00	
	Disengagement from nonpunishment (short-delay)	−0.01	−0.50	
	Disengagement from reward (long-delay)	0.01	0.31	
	Disengagement from nonpunishment (long-delay)	−0.02	−0.70	0.00

Note: R² final model = 0.52**, Adjusted R² = 0.52.

* p < 0.05.

** p < 0.01.

ª The sample size reported reflects the weighted sample size.

^b^ 0 = Female, 1 = Male.

### Post-hoc analysis

That reward biases did not contribute to the increase in substance use over three years was unexpected. This could indicate that these reward biases are not involved in the increase of existing substance use, but in initial use. The incidence of alcohol, tobacco, and cannabis use at baseline was too high to explore this possibility. In contrast, only 5.5% of the adolescents in the current sample had used illicit drugs other than cannabis at baseline, while 13.4% had used these drugs three years later. We first tested the predictive value of attentional biases at T3 on illicit drug use (user/nonuser) at T4 by performing a logistic regression analysis in the subsample of non-illicit (other than cannabis) drug users at T3. The results showed that none of the attentional bias scores, but only substance use (OR = 5.25, Wald = 56.37, p <0.01) at T3 could predict whether one would start using illicit drug in between T3 and T4. We therefore selected the adolescents who started using illicit drugs in between baseline and follow-up (*n* = 53), and examined the prospective relationship between the strength of reward-related biases and the level of subsequent illicit drug use to shed light on the relationship between reward biases and substance use in the initiation stage. First, we performed a bivariate correlation analysis including age, gender, substance use T3, all engagement and disengagement scores and illicit drug use at follow-up. The correlational analysis showed that gender, age, and engagement toward nonpunishment in the long-delay trials were positively related to illicit drug use at follow-up. Subsequently, we conducted a regression analysis in the prediction of level of illicit drug use three years later and included gender, age and substance use at T3 in step 1, and engagement toward nonpunishment in the long-delay trials in step 2. This full model explained 30% (*R²* adjusted = 0.24, *F*(4, 48) = 5.20, *p* <0.01) of all variance (Table 5), and showed that age and engagement toward nonpunishment in the long-delay trials explained unique variance. That is, within the group of adolescents who started using illicit drugs in between both measures those who were older, and those who showed stronger engagement toward longer presented cues of nonpunishment reported a higher level of illicit drug use. Because of the skewed distribution of illicit drug use, we log10 transformed this variable, and repeated the analysis with this transformed variable as the dependent variable in the regression model. The results of the analysis were comparable to the original analysis. We therefore chose to report only the original analysis.

**Table 5:** Hierarchical regression model for variables explaining prospective illicit drug use (amphetamine, cocaine, magic mushrooms) in adolescents who started using illicit drugs in between baseline and follow-up (N = 52^a^).

Variable		Beta	T	R² Change
Step 1				
	(Constant)		−2.72**	
	Gender ª	0.31*	2.44	
	Age	0.35**	2.78	0.24
	Substance Use T3	0.21	1.68	
Step 2				
	(Constant)		-3.00**	
	Gender ^b^	0.25	2.05*	
	Age	0.37**	3.05	
	Substance Use T3	0.12	0.97	
	Engagement toward nonpunishment (long-delay)	0.27*	2.11	0.07

Note: R² final model = 0.30**, Adjusted R² = 0.24

* p < 0.05.

** p < 0.01.

^a^ The sample size reported reflects the weighted sample size.

^b^ 0 = Female, 1 = Male.

## Discussion

Previous cross-sectional research has shown that adolescent substance use is related to a relatively strong automatic engagement toward nonpunishment and a relatively strong voluntary engagement toward reward [12]. As an important next step, the present study tested whether these reward-related attentional biases would also show a predictive relationship with adolescent substance use at a three-year follow-up. This relationship was tested in a large representative cohort of adolescents. The criterion validity of reward-related attentional biases was supported with results showing that a relatively strong automatic engagement toward nonpunishment and a relatively strong voluntary engagement toward reward correlated with adolescent substance use at three-year follow-up. However, the findings did not support the idea that reward-related attentional biases predict changes in substance use behavior. This lead to the idea that these reward biases might not be involved in the increase, but specifically in the initiation phase of substance use. The post-hoc analysis that was restricted to the subgroup of adolescents who started using illicit drugs (other than cannabis) in the three years following the assessment of the biases provided preliminary supportive evidence for reward-related attentional bias as a predictor of future drug use. Stronger voluntary engagement toward nonpunishment showed independent predictive value for the prospective level of illicit drug use.

This study therefore leads to three main findings. First of all, it extends the previous finding that adolescent substance use is related to relatively strong preferential orienting of the attention toward cues of reward and nonpunishment [12]. That is, this study elongated the previous study by showing that reward bias was not only correlated with substance use at the same time, but also with substance use at three years follow up.

Second, the appetitive attentional biases that were correlated with substance use represented the attentional process of enhanced engagement to reward and nonpunishment related cues, instead of a difficulty to disengage from these cues. It seems therefore that adolescent substance use is characterized by preferential orienting of the attention, but not by a difficulty to redirect attention away from appetitive cues. However, there is no straightforward explanation for the apparent differential involvement of short-delay vs. long-delay trials in the engagement scores for alcohol, tobacco, and cannabis use. Especially because the results showed differential patterns related to the use of different substances. We can only speculate about the differential predictive value of more automatic and more controlled attentional engagement. One possibility for this pattern could be that a strong automatic engagement toward nonpunishment relative to punishment reflects a weak automatic tendency to attend to negative consequences (e.g., fear of getting a hang-over), and that a strong voluntary engagement toward reward relative to nonreward represents a heightened voluntary tendency to attend to positive outcomes (i.e., attaining a pleasant feeling after substance use). Further, a strong voluntary engagement toward nonpunishment relative to punishment could reflect a weak voluntary tendency to attend to negative consequences. However, to reach a conclusive explanation, it is needed to replicate and test further these indexes related to substance use.

Finally, we found no predictive involvement of appetitive bias in the increase in substance use in adolescents who already started using substances. A post-hoc analysis did support a prognostic relationship between appetitive bias and level of future substance use in those who initiated the use of a substance (i.e., drugs) after the baseline assessment. These findings give rise to the view that perhaps appetitive bias is especially important in the initiation stages of adolescent substance use. Such a view would be in line with the idea that a heightened sensitivity for stimuli that signal unconditioned reward and relief from punishment [14,15], might predict the development of substance (ab)use. From this perspective, high attentional sensitivity to reward-related stimuli might be a risk factor for heavy initial use, and other factors for the further development and persistence of substance use once substance use behavior has reached a certain level.

As a more critical test of the relevance of attentional bias for the start of using substances such as alcohol, nicotine, and cannabis, it would be important to assess reward-related attentional bias in an even younger sample before they start using these substances. This would give the opportunity to investigate whether reward-related attentional biases precede the initiation of adolescent substance use. In addition, it would be important for future research to examine the effects of modifying attentional bias away from reward cues on subsequent substance use (cf. [26] [27]), as this would provide important information regarding the causal status of reward bias.

Several limitations of the study should be taken into account when interpreting the results. Perhaps most important, it should be acknowledged that the effect-sizes of the predictive relationships were rather small (i.e., *R²* adj = 0.03). Nevertheless, given the relatively small range in substance use in the sample, together with the methodological restrictions of the behavioral measure used (i.e., reaction time measures such as the SOT provide only a rough indicator of the actual attentional processes), small effects are also noteworthy. Further, the importance of small effects in this research area is underscored by the considerable risks for negative health and social consequences of substance use behavior. From this perspective, the convergence of findings between the current study and previous research [12] suggests a reliable relation between attentional bias for reward and substance use that may serve as a potentially useful point of prevention or intervention. A related limitation is, that although the RT’s scores showed good internal consistency, attentional bias scores showed only low to medium split-half correlations. However, for a good understanding of the reliability of this measure, also a test-retest reliability has to be performed. Nevertheless, the interpretations of the SOT scores have to be taken with caution, and more studies are needed to examine the psychometric properties of this measure. An additional point of consideration is that participants might not have been entirely honest in reporting their substance use. Yet, self-report measures of substance use have been found to be valid and reliable as long as confidentiality and anonymity is guaranteed [28] as was the case in the present study. Lastly, the performance measure was one of the last in a longer sequence of behavioral tasks, and fatigue might have influenced participants’ performance.

In summary, consistent with the view that a generally enhanced attentional bias for appetitive cues may set adolescents at risk for developing excessive substance use, this study showed that enhanced attentional engagement toward cues of reward and nonpunishment was associated with adolescent substance use three years later. Although reward-related biases showed no predictive value for an increase in use between middle and late adolescence, a post-hoc analysis provided first evidence that reward biases do have predictive value for the level of illicit drug use for those who started using in the follow-up period. As a more critical test of the relevance of attentional bias in the overall initiation of substance use it would be important to test reward-related attentional bias in an even younger sample, in which participants have not already started using addictive substances. Another interesting next step would be to follow an experimental approach designed to reduce reward bias [29], and to test whether such manipulation would prevent the initiation or reduce the level of substance use in adolescents. If so, this would not only provide more direct support for the (causal) role of reward bias in adolescent substance use, but also a fresh theory-derived clinical tool to prevent the development of substance abuse and addiction.

## Supporting Information

S1 Table Items and response categories of self-reported substance use, subdivided by substance (alcohol, tobacco, cannabis, illegal drugs). Note: Cronbach’s alpha alcohol items: baseline = 0.87, follow-up = 0.86, Cronbach’s alpha tobacco items: baseline = 0.92, follow-up = 0.93, Cronbach’s alpha cannabis items: baseline = 0.92, follow-up = 0.89, Cronbach’s alpha illegal drug items: baseline = 0.54, follow-up = 0.58. Cronbach’s alpha alcohol, tobacco and cannabis items: baseline = 0.69, follow-up = 0.62.
(PDF)

## References

[1] Monshouwer K, Verdurmen JEE, van Dorsselaer SAFM, Smit E, Gorter AF, Vollebergh WAM (2008) Jeugd en riskant gedrag 2007. (Adolescents and risk-taking behavior 2007) Utrecht: TRIMBOS (Netherlands Institute of Mental Health and Addiction). 187 p.

[2] Winters KC, Lee CS (2008) Likelihood of developing an alcohol and cannabis use disorder during youth: Association with recent use and age. Drug Alcohol Depend 92: 239–247. 17888588 10.1016/j.drugalcdep.2007.08.005 PMC2219953

[3] DeWit DJ, Adlaf EM, Offord DR, Ogborne AC (2000) Age at first alcohol use: A risk factor for the development of alcohol disorders. American Journal of Psychiatry 157: 745 10784467 10.1176/appi.ajp.157.5.745

[4] Lynskey MT, Heath AC, Bucholz KK, Slutske WS, Madden PAF, Nelson EC, et al (2003) Escalation of drug use in early-onset cannabis users vs co-twin controls. JAMA 289: 427–433. 12533121 10.1001/jama.289.4.427

[5] Grant BF, Stinson FS, Harford TC (2001) Age at onset of alcohol use and DSM-IV alcohol abuse and dependence: A 12-year follow-up. J Subst Abuse 13: 493–504. 11775078 10.1016/s0899-3289(01)00096-7

[6] Franken IHA (2003) Drug craving and addiction: Integrating psychological and neuropsychopharmacological approaches. Prog Neuropsychopharmacol Biol Psychiatry 27: 563–579. 12787841 10.1016/S0278-5846(03)00081-2

[7] Robinson TE, Berridge KC (1993) The neural basis of drug craving: An incentive- sensitization theory of addiction. Brain Res Rev 18: 247–291. 8401595 10.1016/0165-0173(93)90013-p

[8] Robinson TE, Berridge KC (2001) Incentive-sensitization and addiction. Addiction 96: 103–114. 11177523 10.1046/j.1360-0443.2001.9611038.x

[9] Field M, Cox WM (2008) Attentional bias in addictive behaviors: A review of its development, causes, and consequences. Drug Alcohol Depend 97: 1–20. 10.1016/j.drugalcdep.2008.03.030 18479844

[10] Lubman DI, Peters LA, Mogg K, Bradley BP, Deakin JFW (2000) Attentional bias for drug cues in opiate dependence. Psychological Medicine: A Journal of Research in Psychiatry and the Allied Sciences 30: 169–175. 10.1017/s0033291799001269 10722187

[11] Colder CR, O'Connor R (2002) Attention bias and disinhibited behavior as predictors of alcohol use and enhancement reasons for drinking. Psychology of Addictive Behaviors 16: 325–332. 12503905

[12] van Hemel-Ruiter ME, de Jong PJ, Oldehinkel AJ, Ostafin BD (2013) Reward-related attentional biases and adolescent substance use: The TRAILS study. Psychology of Addictive Behaviors 27: 142–150. 10.1037/a0028271 22564203

[13] Derryberry D, Reed MA (1994) Temperament and attention: Orienting toward and away from positive and negative signals. J Pers Soc Psychol 66: 1128–1139. 8046580 10.1037//0022-3514.66.6.1128

[14] Gray JA (1970) The psychophysiological basis of introversion-extraversion. Behav Res Ther 8: 249–266. 5470377 10.1016/0005-7967(70)90069-0

[15] Gray JA (1982) The neuropsychology of anxiety: An enquiry into the functions of the septo-hippocampal system New York, NY US: Clarendon Press/Oxford University Press.

[16] Derryberry D, Reed MA (2002) Anxiety-related attentional biases and their regulation by attentional control. J Abnorm Psychol 111: 225–236. 12003445 10.1037//0021-843x.111.2.225

[17] Pratt NL (2009) The influence of motivation and reward on selective visual attention. Dissertation Abstracts International: Section B: The Sciences and Engineering 69.

[18] Knyazev GG (2004) Behavioural activation as predictor of substance use: Mediating and moderating role of attitudes and social relationships. Drug Alcohol Depend 75: 309–321. 15283952 10.1016/j.drugalcdep.2004.03.007

[19] Lopez-Vergara H, Colder CR, Hawk LWJ, Wieczorek WF, Eiden RD, Lengua LJ, et al (2012) Reinforcement sensitivity theory and alcohol outcome expectancies in early adolescence. Am J Drug Alcohol Abuse 38: 130–134. 10.3109/00952990.2011.643973 22220630 PMC4033694

[20] O'Connor RM, Colder CR (2005) Predicting alcohol patterns in first-year college students through motivational systems and reasons for drinking. Psychology of Addictive Behaviors 19: 10–20. 15783273 10.1037/0893-164X.19.1.10

[21] Pardo Y, Aguilar R, Molinuevo B, Torrubia R (2007) Alcohol use as a behavioural sign of disinhibition: Evidence from J.A. gray's model of personality. Addict Behav 32: 2398–2403. 17407802 10.1016/j.addbeh.2007.02.010

[22] Huisman M, Oldehinkel AJ, de Winter A, Minderaa RB, de Bildt A, Huizink A, et al (2008) Cohort profile: The dutch 'TRacking adolescents' individual lives' survey'; TRAILS. Int J Epidemiol 37: 1227–1235. 10.1093/ije/dym273 18263649

[23] de Winter AF, Oldehinkel AJ, Veenstra R, Brunnekreef JA, Verhulst FC, Ormel J (2005) Evaluation of non-response bias in mental health determinants and outcomes in a large sample of pre-adolescents. Eur J Epidemiol 20: 173–181. 15792285 10.1007/s10654-004-4948-6

[24] Nederhof E, Jorg F, Raven D, Veenstra R, Verhulst F, Ormel J, et al (2012) Benefits of extensive recruitment effort persist during follow-ups and are consistent across age group and survey method. the TRAILS study. BMC Medical Research Methodology 12: 93 10.1186/1471-2288-12-93 22747967 PMC3585928

[25] Huizink AC, Ferdinand RF, Ormel J, Verhulst FC (2006) Hypothalamic-pituitary-adrenal axis activity and early onset of cannabis use. Addiction 101: 1581–1588. 17034437 10.1111/j.1360-0443.2006.01570.x

[26] Fadardi JS, Cox WM (2009) Reversing the sequence: Reducing alcohol consumption by overcoming alcohol attentional bias. Drug Alcohol Depend 101: 137–145. 10.1016/j.drugalcdep.2008.11.015 19193499

[27] Schoenmakers TM, de Bruin M, Lux IFM, Goertz AG, van Kerkhof DHAT, Wiers RW (2010) Clinical effectiveness of attentional bias modification training in abstinent alcoholic patients. Drug Alcohol Depend 109: 30–36. 10.1016/j.drugalcdep.2009.11.022 20064698

[28] Del Boca F, Darkes J (2003) The validity of self-reports of alcohol consumption: State of the science and challenges for research. Addiction 98: 1–12. 14984237 10.1046/j.1359-6357.2003.00586.x

[29] Houben K, Havermans RC, Nederkoorn C, Jansen A (2012) Beer à no-go: Learning to stop responding to alcohol cues reduces alcohol intake via reduced affective http://www.ncbi.nlm.nih.gov/pubmed/22296168 22296168 10.1111/j.1360-0443.2012.03827.x

