# Supplementary material for: Reward-Related Attentional Bias and Adolescent Substance Use: A Prognostic Relationship?
Source: PLoS One. 2015 Mar 27;10(3):e0121058. doi: 10.1371/journal.pone.0121058 (PMC4376386; doi:10.1371/journal.pone.0121058)
Supplement: S1 Table — Note: Cronbach’s alpha alcohol items: baseline = 0.87, follow-up = 0.86, Cronbach’s alpha tobacco items: baseline = 0.92, follow-up = 0.93, Cronbach’s alpha cannabis items: baseline = 0.92, follow-up = 0.89, Cronbach’s alpha illegal drug items: baseline = 0.54, follow-up = 0.58. Cronbach’s alpha alcohol, tobacco and cannabis items: baseline = 0.69, follow-up = 0.62. (PDF) [file pone.0121058.s001.pdf]

Item	Substance	Question	Response categories
1	Alcohol	How many times did you drink alcohol in your lifetime?	1-10 = 1 to 10 times, 11 = 11-19 times, 12 = 20-39 times, 13 = 40 times or more
2		How many times did you drink alcohol in the last twelve months?	1-10 = 1 to 10 times, 11 = 11-19 times, 12 = 20-39 times, 13 = 40 times or more
3		How many times did you drink alcohol in the last 4 weeks?	1-10 = 1 to 10 times, 11 = 11-19 times, 12 = 20-39 times, 13 = 40 times or more
4		At how many week days do you normally drink alcohol?	0-3 = 0 to 3 days
5		How many glasses of alcohol do you normally drink at a week day?	0-6 = 0 to 6 glasses, 7 = 7-10 glasses, 8 = 11 or more glasses per day
6		At how many weekend days do you normally drink alcohol?	0-3 = 0 to 3 days
7		How many glasses of alcohol do you normally drink at a weekend day?	0-6 = 0 to 6 glasses, 7 = 7-10 glasses, 8 = 11 or more glasses per day
8	Tobacco	Did you ever smoke, even if it was just one cigarette or a few drafts?	0 = never, 1 = 1 or 2 times, 2 = not every day, 3 = I stopped, 4 = every day
9		How many cigarettes do you normally smoke at a smoking day?	Continuous, 0 - ∞
10		How many cigarettes did you smoke in the past four weeks?	0 = I never smoke, 1 = 0 cigs, 2 = less than 1, 3= 1-5 cigs, 4 = 6-10 cigs, 5 = 11-20, 6 = 20 or more
11	Cannabis	How many times did you use weed or hash in your lifetime?	1-10 = 1 to 10 times, 11 = 11-19 times, 12 = 20-39 times, 13 = 40 times or more
12		How many times did you use weed or hash in the last twelve months?	1-10 = 1 to 10 times, 11 = 11-19 times, 12 = 20-39 times, 13 = 40 times or more
13		How many times did you use weed or hash in the last four weeks?	1-10 = 1 to 10 times, 11 = 11-19 times, 12 = 20-39 times, 13 = 40 times or more
14	Illegal drugs	How many times did you use amphetamine?	1-10 = 1 to 10 times, 11 = 11-19 times, 12 = 20-39 times, 13 = 40 times or more
15		How many times did you use cocaine?	1-10 = 1 to 10 times, 11 = 11-19 times, 12 = 20-39 times, 13 = 40 times or more
16		How many times did you use magic mushrooms?	1-10 = 1 to 10 times, 11 = 11-19 times, 12 = 20-39 times, 13 = 40 times or more

Note: Cronbach's alpha alcohol items: baseline = 0.87, follow-up = 0.86, Cronbach's alpha tobacco items: baseline = 0.92, follow-up = 0.93, Cronbach's alpha cannabis items: baseline = 0.92, follow-up = 0.89, Cronbach's alpha illegal drug items: baseline = 0.54, follow-up = 0.58. Cronbach's alpha alcohol, tobacco and cannabis items: baseline = 0.69, follow-up = 0.62.
